# 1535. Harm Reduction: A Missing Piece in Holistic ID Care for Patients Who Inject Drugs

**DOI:** 10.1093/ofid/ofac492.090

**Published:** 2022-12-15

**Authors:** Sarah M Fracasso Francis, Susan E Beekmann, Phillip M Polgreen, Laura R Marks, Stephen Y Liang, Michael J Durkin, Nathanial S Nolan

**Affiliations:** Washington University in St. Louis School of Medicine, Saint Louis, Missouri; University of Iowa, IOWA CITY, Iowa; University of Iowa, IOWA CITY, Iowa; Washington University in St. Louis, St Louis, Missouri; Washington University School of Medicine, St Louis, Missouri; Washington University School of Medicine, St Louis, Missouri; Washington University in St. Louis, St Louis, Missouri

## Abstract

**Background:**

The rise in injection drug use (IDU) has led to an increase in drug-related infections and hospitalizations. Harm reduction is an important strategy for preventing infections among people who inject drugs (PWID). We set out to evaluate how infectious disease physicians counsel PWID with serious injection-related infections on harm reduction practices.

**Methods:**

An electronic survey was distributed to 1,510 physician members of the Emerging Infectious Network (EIN) to determine which harm reduction principles ID physicians recommend to PWID to reduce infection risks. EIN members who do not care for PWID were excluded. Two weekly reminders were sent to increase the response rate.

**Results:**

Three hundred ninety-nine (26%, still in progress) ID physicians responded to the survey. Practice settings included: university (37%), community (27%), non-university teaching (23%), or other (14%) hospital settings. Of those, 279 (70%) reported routinely caring for PWID. 274 (98%) performed screening for HIV and hepatitis, and 239 (86%) discussed the risk of these viral infections. 65% prescribed immunization against hepatitis and 44% discussed HIV PrEP. 57% (n=159) reported not counseling patients on safer injection strategies. Common reasons for not counseling included limited time and a desire to emphasize antibiotic therapy/medical issues (33%, n=91), lack of training (30%, n=85), and believing that it would be better addressed by other services (27%, n=75). Among physicians who reported counseling PWID, they recommended abstinence from IDU (38%, n=105), handwashing & skin cleansing prior to injection (33%, n=92), avoiding injecting areas of skin breakdown (27%, n=76), and cleaning needles between use (26%, n= 73). Finally, 17% (N=47) reported no access to any addiction services.

**Conclusion:**

Almost all ID physicians screen PWID for HIV and viral hepatitis and discuss the risks of these infections. Despite frequently encountering PWID, less than half of ID physicians provide safer injection advice. Opportunities exist to standardize education of PWID on harm reduction, emphasizing safer injection practices in conjunction with other strategies (e.g. HIV PrEP, HAV/HBV vaccination) to prevent infections.

**Disclosures:**

**All Authors**: No reported disclosures.

